# The Role of Genetically Determined Glycemic Traits in Breast Cancer: A Mendelian Randomization Study

**DOI:** 10.3389/fgene.2020.540724

**Published:** 2020-09-16

**Authors:** Su Yon Jung, Nicholas Mancuso, Sihao Han, Zuo-Feng Zhang

**Affiliations:** ^1^Translational Sciences Section, Jonsson Comprehensive Cancer Center, School of Nursing, University of California, Los Angeles, Los Angeles, CA, United States; ^2^Division of Biostatistics, Department of Preventive Medicine, Keck School of Medicine, University of Southern California, Los Angeles, CA, United States; ^3^Center for Genetic Epidemiology, Keck School of Medicine, University of Southern California, Los Angeles, CA, United States; ^4^Norris Comprehensive Cancer Center, University of Southern California, Los Angeles, CA, United States; ^5^Department of Epidemiology, Fielding School of Public Health, University of California, Los Angeles, Los Angeles, CA, United States; ^6^Center for Human Nutrition, David Geffen School of Medicine, University of California, Los Angeles, Los Angeles, CA, United States

**Keywords:** genetically determined glucose and insulin, breast cancer, Mendelian randomization, obesity, diabetes

## Abstract

**Background:**

Circulating glycemic traits (GTs) have been considered a risk factor for breast cancer, but studies using GT-associated genetic variants as an instrumental variable are limited and inconclusive.

**Methods:**

Our Mendelian Randomization analysis used the most recent genome-wide datasets focusing on European women.

**Results:**

Of 44 single-nucleotide polymorphisms (SNPs) with GTs, 38 fasting-glucose and 6 fasting-insulin SNPs showed heterogeneous associations with breast cancer, without significant directional pleiotropy observed.

**Conclusion:**

Our findings indicate a null association between genetically determined GTs and breast cancer risk among European women. Our findings may contribute to more complete characterizing of metabolic pathways in GTs and breast cancer.

## Introduction

Previous studies for circulating glycemic traits (GTs), including fasting glucose (FG) and insulin (FI) concentrations, have shown inconsistent associations with breast cancer development (Gunter et al., 2009; Sieri et al., 2012; Boyle et al., 2013; Hernandez et al., 2014). This is partially due to selection bias, confounding, short time exposure to such metabolic biomarkers, measurement errors, and reverse causation. We tried to address those challenges by using a 2-sample Mendelian Randomization (MR) approach and examined whether genetically determined GTs are causally associated with breast cancer risk. The MR method may provide a relatively unbiased causal relationship between phenotype and cancer outcome because it reduces potential bias and confounding and prevents reverse causation by the random assortment of alleles at meiosis, resulting in random assignment of exposure, which precedes the phenotype and clinical outcomes (Merino et al., 2017).

## Materials and Methods

For the GT instrumental variables, we used the recently updated publicly available data in 2019 from genome-wide association studies (GWASs) of the Meta-Analysis of Glucose and Insulin-related traits Consortium (MAGIC) in non-diabetic European women^1^. Detailed rationale and design of the studies have been described elsewhere (Scott et al., 2012). For breast cancer outcomes, we pulled 4 datasets from 2 independent consortia: Breast Cancer Association Consortium (BCAC^2^): (i) OncoArray; the Atlas of GWAS Summary Statistics (ATLAS^3^): (ii) CGEMS Breast Cancer GWAS, (iii) GEE adjusted for age, and (iv) GEE for age and body mass index. Study participants from each dataset provided written informed consent. Genetic instruments for each dataset were single-nucleotide polymorphisms (SNPs) associated with the trait at the genome-wide level (p < 5E-08).

We performed MR analysis using the inverse-variance weighted method (Burgess et al., 2013) which quantifies the genetically determined association between GTs and breast cancer risk. We assume that summarized data are available for multiple genetic variants in relation to the risk factor of interest X and the outcome Y; genetic variant k, k = 1,…, K is associated with an observed X_k_ mean change in the risk factor per additional variant allele with standard error σ_Xk_ and an observed Y_k_ changes in the log-odds or the log-probability of an outcome per allele with standard error σ_Yk_. The inverse-variance weighted estimates combine the ratio estimates from each variant in a fixed-effect meta-analysis model:

$$\overset{\wedge }{\underset{I\,V\,W}{\beta }}=\frac{\sum_{K}X_{k}\,Y_{k}\,\sigma_{y\,k}^{-2}}{\sum_{k}X_{k}^{2}\,\sigma_{Y\,k}^{-2}}$$

The approximate standard error of the estimate is:

$$se(\overset{\wedge }{\underset{I\,V\,W}{\beta }})=\sqrt{\frac{1}{\sum_{k}X_{k}^{2}\,\sigma_{Y\,k}^{-2}}}$$

The results were reported as risk ratios and 95% confidence intervals for the change in breast cancer risk per unit increase in FG (mmol/L) or natural log-transformed FI (pmol/L). To determine the extent of pleiotropic signal, we applied Cochran’s Q test and the MR-Egger analysis. Given obesity and diabetes’s established role for breast cancer, we excluded those relevant SNPs from the analysis. R3.6.1 was used. The Institutional Review Board of the University of California, Los Angeles, approved this study.

## Results and Discussion

**FIGURE 1:**
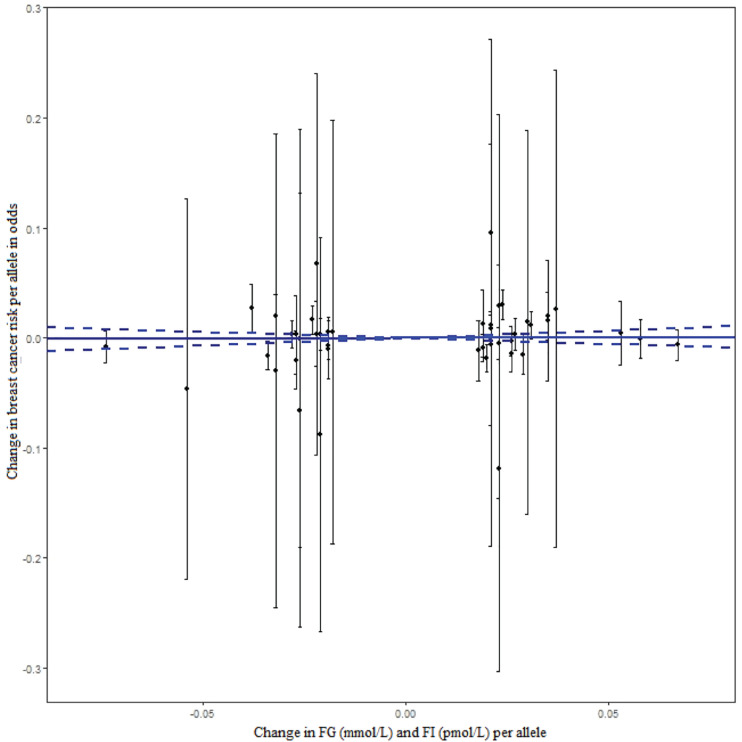
The effect of individual genetic instrumental variables for GTs on breast cancer risk. Each black dot represents a genome-wide GT-associated genetic variant. The blue lines indicate regression and 95% CIs of GTs on breast cancer risk (OR = 1.014, 95% CI: 0.895–1.147). CI, confidence interval; FG, fasting glucose; FI, fasting insulin; OR, odds ratio; GTs, glycemic traits.

Of 430 GWA-based SNPs related to GTs in MAGIC, 44 SNPs within linkage disequilibrium (*r*^2^ < 0.1) were matched to either BCAC or ATLAS datasets (Table 1). The 38 FG-SNPs overall and stratified by cancer data source showed heterogeneous results but mostly showed a slightly increased effect on breast cancer risk without reaching statistical significance (Table 2 and Figure 1). After excluding top GWA-SNPs associated with type 2 diabetes and visceral obesity, the directions of the associations between GTs and breast cancer were changed in *OncoArray* but not in *ATLAS-CGEMS.* The 6 FI-SNPs showed similar patterns for the associations with breast cancer. No significant directional pleiotropy was observed (Table 3).

**TABLE 1:** Top GWA SNPs associated with glucose-metabolism phenotypes and breast cancer risk.

Gene*	SNP	Chr	Position	Allele	Alt allele frequency	Phenotype^†^		Breast cancer¥		
							
				Ref/Alt		Effect size	p	OR	95% CI	p
**Fasting glucose Breast cancer: OncoArray**										
PROX1	**rs340874**	**1**	**214159256**	**T/C**	0.562	**0.020**	**1.69E-13**	**0.982**	**(0.969–0.994)**	**0.004**
G6PC2	**rs560887**	**2**	**169763148**	**T/C**	0.674	**0.067**	**8.08E-92**	0.994	(0.980–1.008)	0.389
GCKR	**rs780094**	**2**	**27741237**	**T/C**	0.606	**0.031**	**3.06E-26**	1.012	(0.999–1.024)	0.070
ADCY5	**rs11708067**	**3**	**123065778**	**G/A**	0.774	**0.027**	**5.01E-16**	1.004	(0.989–1.018)	0.644
SLC2A2	**rs11924648**	**3**	**170717996**	**G/A**	0.863	**0.029**	**1.74E-11**	0.985	(0.968–1.003)	0.104
PCSK1	**rs7713317**	**5**	**95716722**	**G/A**	0.695	**0.023**	**6.50E-14**	0.995	(0.981–1.009)	0.476
AC006045.3	**rs1558318**	**7**	**15065612**	**T/A**	0.545	−**0.028**	**2.93E-20**	1.003	(0.991–1.016)	0.595
GCK	**rs4607517**	**7**	**44235668**	**G/A**	0.195	**0.058**	**2.66E-46**	1.000	(0.982–1.017)	0.959
SLC30A8	**rs3802177**	**8**	**118185025**	**G/A**	0.239	−**0.034**	**1.12E-27**	**0.984**	**(0.971–0.997)**	**0.016**
PPP1R3B	**rs983309**	**8**	**9177732**	**T/G**	0.903	−**0.032**	**4.85E-13**	**1.020**	**(1.000–1.040)**	**0.050**
GLIS3	**rs10814916**	**9**	**4293150**	**C/A**	0.434	−**0.019**	**7.61E-10**	1.006	(0.993–1.019)	0.372
CDKN2B-AS1	**rs2383208**	**9**	**22132076**	**G/A**	0.792	**0.026**	**1.19E-13**	0.986	(0.970–1.001)	0.070
ADRA2A	**rs11195502**	**10**	**113039667**	**T/C**	0.925	**0.035**	**7.41E-12**	1.020	(0.998–1.042)	0.080
TMEM258	**rs102275**	**11**	**61557803**	**T/C**	0.351	−**0.019**	**1.01E-09**	0.993	(0.981–1.006)	0.292
MTNR1B	**rs10830963**	**11**	**92708710**	**G/C**	0.700	−**0.074**	**1.36E-98**	0.992	(0.978–1.007)	0.284
CTD-2210P24.6	**rs6485644**	**11**	**45855998**	**T/C**	0.531	**0.019**	**1.39E-10**	0.991	(0.979–1.003)	0.154
MADD	**rs7944584**	**11**	**47336320**	**T/A**	0.712	**0.024**	**2.20E-12**	**1.031**	**(1.016–1.045)**	<**0.001**
PDX1	**rs11619319**	**13**	**28487599**	**G/A**	0.788	−**0.021**	**9.26E-10**	1.003	(0.989–1.018)	0.662
VPS13C/C2CD4A/B	**rs4502156**	**15**	**62383155**	**T/C**	0.420	−**0.023**	**3.07E-15**	**1.016**	**(1.003–1.030)**	**0.014**
**FG, Breast cancer: ATLAS-CGEMS**										
PROX1	rs340874	**1**	**214159255**	**T/C**	0.562	**0.020**	**1.69E-13**	0.991	(0.819–1.198)	0.913
G6PC2	rs560887	**2**	**169763147**	**T/C**	0.674	**0.067**	**8.08E-92**	1.006	(0.846–1.196)	0.745
GCKR	rs780094	**2**	**27741236**	**T/C**	0.606	**0.031**	**3.06E-26**	0.994	(0.828–1.193)	0.983
RNU1-70P	rs11709140	**3**	**170694496**	**T/C**	0.137	−**0.026**	**1.90E-09**	0.937	(0.769–1.140)	0.435
ADCY5	rs2877716	**3**	**123094450**	**T/C**	0.752	**0.023**	**7.27E-11**	1.029	(0.865–1.225)	0.919
PCSK1	rs4869272	**5**	**95539447**	**T/C**	0.323	−**0.022**	**1.64E-13**	1.070	(0.900–1.272)	0.721
AC006045.3	rs2191348	**7**	**15064254**	**T/G**	0.482	−**0.026**	**2.56E-18**	1.000	(0.827–1.208)	1.000
GCK	rs4607517	**7**	**44235667**	**G/A**	0.195	**0.058**	**2.66E-46**	0.918	(0.762–1.105)	0.207
SLC30A8	rs13266634	**8**	**118184782**	**T/C**	0.761	**0.030**	**6.72E-21**	1.015	(0.852–1.208)	0.467
PPP1R3B	rs983309	**8**	**9177731**	**T/G**	0.903	−**0.032**	**4.85E-13**	0.971	(0.783–1.203)	0.937
GLIS3	rs10814916	**9**	**4293149**	**C/A**	0.434	−**0.019**	**7.61E-10**	0.999	(0.823–1.213)	0.999
CDKN2B-AS1	rs2383208	**9**	**22132075**	**G/A**	0.792	**0.026**	**1.19E-13**	**1.065**	**(0.886–1.278)**	**0.039**
BTBD7P2	rs4258313	**10**	**113032397**	**T/G**	0.914	**0.037**	**1.82E-11**	1.027	(0.827–1.275)	0.625
TMEM258	rs102275	**11**	**61557802**	**T/C**	0.351	−**0.019**	**1.01E-09**	1.029	(0.865–1.225)	0.768
CRY2	rs11607883	**11**	**45839708**	**G/A**	0.469	−**0.018**	**1.86E-10**	1.006	(0.829–1.219)	0.967
ACP2	rs11988	**11**	**47261259**	**G/A**	0.372	−**0.021**	**5.07E-12**	0.916	(0.765–1.095)	0.412
MTNR1B	rs1387153	**11**	**92673827**	**T/C**	0.728	−**0.054**	**1.27E-58**	0.954	(0.803–1.134)	0.619
PDX1-AS1	rs2293941	**13**	**28491197**	**G/A**	0.212	**0.021**	**1.42E-09**	1.101	(0.923–1.312)	0.470
NPM1P47	rs7172432	**15**	**62396388**	**G/A**	0.580	**0.023**	**3.15E-11**	0.888	(0.739–1.068)	0.448
**FG, Breast cancer: ATLAS-GEEA**										
PROX1-AS1	rs1431985	**1**	**214148245**	**G/A**	0.327	−**0.019**	**1.15E-09**	0.989	(0.964–1.016)	0.427
SNX17	rs1528533	**2**	**27595755**	**G/C**	0.458	**0.018**	**5.29E-09**	0.988	(0.962–1.016)	0.406
ABCB11	rs494874	**2**	**169789305**	**T/C**	0.628	**0.053**	**1.82E-68**	1.004	(0.976–1.034)	0.771
SLC2A2	rs10513686	**3**	**170725541**	**G/A**	0.142	−**0.027**	**3.73E-10**	1.003	(0.968–1.039)	0.878
AC006045.3	rs10487796	**7**	**15063429**	**T/A**	0.525	−**0.027**	**8.54E-20**	0.980	(0.954–1.007)	0.140
BTBD7P2	rs10509938	**10**	**113028616**	**T/C**	0.920	**0.035**	**3.18E-11**	1.016	(0.961–1.073)	0.580
MADD	rs10501320	**11**	**47293798**	**G/C**	0.292	−**0.022**	**3.47E-08**	1.004	(0.975–1.034)	0.808
MTNR1B	rs1387153	**11**	**92673827**	**T/C**	0.728	−**0.054**	**1.27E-58**	1.020	(0.987–1.054)	0.230
FADS2	rs1535	**11**	**61597971**	**G/A**	0.659	**0.019**	**2.75E-09**	1.013	(0.983–1.044)	0.405
**FG, Breast cancer: ATLAS-GEEAB**										
PROX1-AS1	rs1431985	**1**	**214148245**	**G/A**	0.327	−**0.019**	**1.15E-09**	0.990	(0.964–1.016)	0.441
SNX17	rs1528533	**2**	**27595755**	**G/C**	0.458	**0.018**	**5.29E-09**	0.988	(0.961–1.016)	0.399
ABCB11	rs494874	**2**	**169789305**	**T/C**	0.628	**0.053**	**1.82E-68**	1.004	(0.975–1.033)	0.801
SLC2A2	rs10513686	**3**	**170725541**	**G/A**	0.142	−**0.027**	**3.73E-10**	1.001	(0.966–1.038)	0.941
AC006045.3	rs10487796	**7**	**15063429**	**T/A**	0.525	−**0.027**	**8.54E-20**	0.980	(0.954–1.007)	0.146
BTBD7P2	rs10509938	**10**	**113028616**	**T/C**	0.920	**0.035**	**3.18E-11**	1.014	(0.959–1.072)	0.624
MADD	rs10501320	**11**	**47293798**	**G/C**	0.292	−**0.022**	**3.47E-08**	1.004	(0.975–1.034)	0.798
MTNR1B	rs1387153	**11**	**92673827**	**T/C**	0.728	−**0.054**	**1.27E-58**	1.020	(0.987–1.055)	0.233
FADS2	rs1535	**11**	**61597971**	**G/A**	0.659	**0.019**	**2.75E-09**	1.013	(0.982–1.044)	0.413
**Fasting insulin Breast cancer: OncoArray**										
COBLL1	rs10179126	**2**	**165511794**	**G/C**	0.605	**0.021**	**3.78E-08**	1.008	(0.995–1.021)	0.208
GCKR	rs780093	**2**	**27742603**	**T/C**	0.606	**0.021**	**8.48E-09**	1.011	(0.999–1.024)	0.076
ZNF12/AC073343.13	rs7798471	**7**	**6744957**	**T/C**	0.243	**0.026**	**1.55E-08**	0.997	(0.984–1.011)	0.680
RP11-115J16.1	rs4240624	**8**	**9184231**	**G/A**	0.925	−**0.038**	**1.10E-09**	**1.027**	**(1.005–1.050)**	**0.016**
**FI, Breast cancer: ATLAS-CGEMS**										
GCKR	rs780094	**2**	**27741236**	**T/C**	0.606	**0.021**	**1.00E-08**	0.994	(0.828–1.193)	0.983
ZNF12/AC073343.13	rs7798471	**7**	**6744956**	**T/C**	0.243	**0.026**	**1.55E-08**	1.063	(0.895–1.263)	0.708
PPP1R3B	rs983309	**8**	**9177731**	**T/G**	0.903	−**0.032**	**2.03E-09**	0.971	(0.783–1.203)	0.937

Alt, alternative allele; Chr, chromosome; CI, confidence interval; CGEMS, Cancer Genetic Markers of Susceptibility Breast Cancer Genome-wide Association (GWA) Study; FG, fasting glucose; FI, fasting insulin; GEEA, generalized estimating equation regression adjusted for age; GEEAB, GEEA additionally adjusted for body mass index; OR, odds ratio; Ref, reference allele; SNP, single-nucleotide polymorphism. Numbers in bold face are statistically significant. *Genes were arranged by GWA data source for breast cancer: OncoArray, ATLAS-CGEMS, ATLAS-GEEA, and ATLAS-GEEAB. ^†^Phenotype includes FG and FI; the relevant top SNPs (p < 5E-08) were identified by MAGIC. ¥The SNPs for association with breast cancer risk were pulled from 2 independent consortia (OncoArray and ATLAS [CGEMS; GEEA; and GEEAB]).

**TABLE 2:** Mendelian randomization analysis for the effect of genetically determined glucose-metabolism phenotypes on risk for breast cancer.

A set of GM-SNPs arranged by breast-cancer data source	SNP	OR	95% CI	p	p_hat_^†^
	n				
**Fasting glucose**					
OncoArray	19	1.002	(0.831–1.209)	0.984	< 0.001
OncoArray*	5	1.045	(0.354–3.081)	0.916	<0.001
OncoArray – T2DM¥	16	0.981	(0.800–1.203)	0.843	<0.001
OncoArray – T2DM¥*	4	0.808	(0.157–4.150)	0.706	<0.001
ATLAS-CGEMS	19	1.146	(0.507–2.592)	0.729	0.993
ATLAS-CGEMS – T2DM¥	16	1.002	(0.400–2.513)	0.996	0.980
ATLAS-GEEA	9	1.034	(0.748–1.429)	0.817	0.640
ATLAS-GEEAB	9	1.029	(0.747–1.416)	0.843	0.664
FG: Pooled MR	38	1.014	(0.889–1.156)	0.830	0.0007
**Fasting insulin**					
OncoArray	4	1.002	(0.417–2.405)	0.995	0.014
OncoArray –WHR¥	3	0.895	(0.202–3.964)	0.779	0.013
ATLAS-CGEMS	3	3.335	(0.147–75.424)	0.238	0.889
FI: Pooled MR	6	1.003	(0.579–1.737)	0.988	0.056

CI, confidence interval; CGEMS, Cancer Genetic Markers of Susceptibility Breast Cancer Genome-wide Association (GWA) Study; FG, fasting glucose; FI, fasting insulin; GEEA, generalized estimating equation regression adjusted for age; GEEAB, GEEA, additionally adjusted for body mass index; GM, glucose metabolism; MR, Mendelian randomization; OR, odds ratio; SNP, single–nucleotide polymorphism; T2DM, type 2 diabetes; WHR, waist-to-hip ratio. ^†^P_hat_ was estimated via Cochran’s Q; by correcting multiple comparisons, the MR results for the following sets of SNPs were statistically heterogeneous: FG-OncoArray; FG-OncoArray*; FG-OncoArray-T2DM¥; FG-OncoArray-T2DM¥*; FG-Pooled MR; and FI-OncoArray-WHR¥. *A subset of the GM-SNPs that are statistically associated with breast cancer (p < 0.05) was included in the analysis. ¥GM-SNPs excluding top GWA-SNPs associated with T2DM or WHR were analyzed to reduce the pleiotropic effect from T2DM or WHR, respectively.

**TABLE 3:** Mendelian randomization–Egger test results^†^.

A set of GM-SNPs arranged by breast-cancer data source	Intercept	95% CI	*p*
**Fasting glucose**			
*OncoArray*	1.000	(0.993–1.007)	0.995
OncoArray*	1.004	(0.967–1.043)	0.743
*OncoArray* – T2DM¥	1.002	(0.994–1.009)	0.607
*OncoArray* – T2DM¥*	1.010	(0.954–1.069)	0.533
*ATLAS-CGEMS*	0.994	(0.966–1.023)	0.683
*ATLAS-CGEMS* – T2DM¥	0.987	(0.956–1.020)	0.414
*ATLAS-GEEA*	0.999	(0.988–1.011)	0.863
*ATLAS-GEEAB*	0.999	(0.988–1.010)	0.830
FG: Pooled MR–Egger	1.000	(0.995–1.004)	0.883
**Fasting insulin**			
*OncoArray*	1.014	(0.995–1.034)	0.088
*OncoArray* – WHR¥	1.014	(0.931–1.103)	0.291
*ATLAS-CGEMS*	1.003	(0.698–1.442)	0.929
FI: Pooled MR–Egger	1.014	(1.005–1.024)	0.015

CI, confidence interval; CGEMS, Cancer Genetic Markers of Susceptibility Breast Cancer Genome-wide Association (GWA) Study; FG, fasting glucose; FI, fasting insulin; GEEA, generalized estimating equation regression adjusted for age; GEEAB, GEEA additionally adjusted for body mass index; GM, glucose metabolism; MR, Mendelian randomization; SNP, single-nucleotide polymorphism; T2DM, type 2 diabetes; WHR, waist-to-hip ratio. ^†^MR–Egger test cannot estimate standard error with a single or 2 SNPs. *A subset of the GM-SNPs that are statistically associated with breast cancer (p < 0.05) was included in the analysis. ¥GM-SNPs excluding top GWA-SNPs associated with T2DM or WHR were analyzed to reduce the pleiotropic effect from T2DM or WHR, respectively.

We analyzed the relatively large and most-updated GWA-datasets for causality between GTs and breast cancer. Given that associations between metabolic markers and breast cancer risk can differ by menopausal status, our findings may be confounded. However, data was not available on the menopausal status, thus warranting future studies that account for this difference. In addition, whereas MR is considered a conservative approach, it may be confounded when modeled SNPs independently affect breast cancer risk through intermediate traits other than GTs.

Our study results should be interpreted with caution because of population structure bias (i.e., results biased due to tagged environmental factors) and unmeasured confounding factors that could have introduced bias. MR analysis might also be subject to non-linearity between exposure and outcome, but potential violation of the linearity assumption tends to bias MR estimates toward the null, rather than generating a spurious association (Smith and Ebrahim, 2003). Moreover, our study may not be generalized to other races or ethnicity, in which the association between genetic instruments, GTs, and breast cancer risk may be different.

Our findings indicate a null association between genetically determined GTs and breast cancer risk among European women. Our study may contribute to more complete characterizing of molecular pathways in GTs and breast cancer. It also highlights the need to conduct a more comprehensive and individual-level analysis using more detailed trait information, including risk causing confusion in this field of research.

## Data Availability Statement

The datasets generated for this study are available on request to the corresponding author.

## Author Contributions

SJ, NM, SH, and Z-FZ designed the study. SJ and SH performed the genomic data QC and statistical analysis and interpreted the data. NM and Z-FZ supervised the genomic data QC and analysis and participated in the study coordination and interpreted the data. SJ secured funding for this project. All authors participated in the manuscript writing and editing, read and approved the submission of the manuscript.

## Conflict of Interest

The authors declare that the research was conducted in the absence of any commercial or financial relationships that could be construed as a potential conflict of interest.

## Footnotes

1 https://www.magicinvestigators.org/

2 http://bcac.ccge.medschl.cam.ac.uk/bcacdata

3 https://atlas.ctglab.nl
